# Multi-scale feature fusion-based vision mamba for robust plant disease image classification on field-acquired plantdoc data

**DOI:** 10.3389/fpls.2026.1842426

**Published:** 2026-06-03

**Authors:** Shanjiang Zhang, Renjing Liu

**Affiliations:** 1 School of Management, Xi’an Jiaotong University, Xi’an, China; 2 Shandong Careman Robot Co., Ltd, Jinan, China

**Keywords:** deep learning, Multi-Scale Feature Fusion, plant disease classification, PlantDoc dataset, precision agriculture, Vision Mamba

## Abstract

**Introduction:**

Existing convolutional neural networks and Transformers cannot effectively capture fine-grained local lesion features and long-range contextual dependencies simultaneously in field-collected plant images. To address this research limitation, we aim to design an effective lightweight model suitable for plant disease identification in complex field scenarios.

**Methods:**

This work proposes an improved Vision Mamba network for plant disease classification based on the challenging PlantDoc dataset. Three dedicated modules are embedded into the framework, including the Multi-Scale Feature Fusion Module (MFFM), Adaptive Channel Attention Mechanism (ACAM) and Lightweight Residual Connection (LRC). The MFFM fuses multi-scale texture, shape and semantic lesion features extracted from shallow, medium and deep network layers. The ACAM adaptively highlights disease-related feature channels and suppresses irrelevant background interference. The LRC structure is adopted to relieve the gradient vanishing problem existing in deep selective state space model (SSM) networks.

**Results:**

Experimental results on the filtered PlantDoc dataset show that the presented model obtains an overall accuracy of 92.67%, macro precision of 91.83%, macro recall of 91.56% and macro F1-score of 91.70% on independent test samples, which outperforms the original Vision Mamba baseline by 5.33% in accuracy. Five-fold stratified cross-validation achieves stable accuracy at 92.41 ± 0.24%, and paired t-tests prove that the performance improvement is statistically significant with p<0.05. Ablation experiments confirm the combined contribution of the three designed modules.

**Discussion:**

Error analysis and confusion matrix visualization reveal that the main classification errors are derived from high similarity among different plant disease categories. This study fully verifies the application potential of state space models in agricultural computer vision tasks. The proposed method can serve as an efficient technical scheme for intelligent identification of crop diseases and is well applicable to edge device deployment in precision agriculture practice.

## 1 Introduction

Precision agriculture has emerged as a core paradigm for the intelligent development of modern agricultural production, with automated plant disease detection and classification serving as a critical technical pillar to ensure crop yield and quality. Timely and accurate identification of plant diseases can reduce excessive application of agrochemicals and minimize economic losses caused by epidemic spread, thereby supporting the transition toward green and efficient agricultural production (Han and Wang, 2025). Traditional plant disease detection relies on manual field scouting and expert morphological diagnosis, a process characterized by low throughput, strong subjectivity, and limited geographic coverage, which cannot satisfy the demand for large-scale, real-time disease monitoring in modern agriculture. Leveraging the rapid advances in computer vision and deep learning, image-based plant disease classification has become the prevailing research direction, as these methods automatically extract disease-relevant visual features and enable efficient, objective identification at scale (Yang et al., 2025).

Field-acquired plant images present unique technical challenges that distinguish them from benchmark datasets such as ImageNet. Complex background interference, including soil, weeds, uneven illumination, and occluding foliage, often obscures disease lesions. The morphological characteristics of plant disease symptoms exhibit high inter-class similarity (e.g., early blight versus leaf spot) and substantial intra-class variability due to developmental stage, cultivar, and environmental stress. Effective classification therefore necessitates the simultaneous capture of fine-grained local lesion features (color, texture, shape) and long-range contextual dependencies linking lesion regions to host-plant organs (Han and Wang, 2025). This dual requirement poses a fundamental architectural challenge that existing models address only partially.

Convolutional neural networks (CNNs) have demonstrated competitive performance in plant disease image classification owing to their powerful local feature extraction capabilities and mature design principles (Riheb et al., 2024; Jiang and Li, 2020). Representative architectures such as DenseNet (Huang et al., 2017) and EfficientNet (Tan and Le, 2019) leverage dense connectivity and compound scaling, respectively, to enhance gradient flow and accuracy. Lightweight variants such as MobileNetV3 (Howard et al., 2019) further enable edge deployment through depth-wise separable convolutions. Nevertheless, the fixed receptive field of convolutional operations inherently limits the modeling of long-range contextual dependencies between spatially discrete lesion regions and plant organs, leading to insufficient feature representation for images with scattered symptoms and cluttered backgrounds (Murphy et al., 2024; González-Briones et al., 2025). Beyond architectural improvements within single backbones, complementary strategies such as cross-species transfer learning and handcrafted feature selection have also been explored. Yan et al. (2023) propose a deep transfer learning framework for cross-species plant disease diagnosis that adapts mixed subdomains between source and target species, demonstrating that domain-adaptive transfer can mitigate performance degradation caused by dataset shift across different plant species. Their work highlights the importance of addressing cross-domain bias when models are deployed across diverse crop types. On the feature engineering side, Xie et al. (2023) develop a salp swarm algorithm for feature selection (SSAFS) that determines the optimal combination of handcrafted features to maximize classification accuracy while minimizing feature dimensionality. Experimental results on six plant phenomics datasets validate that intelligent feature selection can effectively identify the most discriminative descriptors for diseased plant image classification. These studies reveal a common insight: whether through automated deep feature learning or optimized handcrafted feature selection, the core challenge lies in extracting the most informative and robust features from highly variable plant disease imagery. However, transfer learning approaches depend heavily on the availability of large-scale labeled source data, while feature selection methods require careful design of handcrafted descriptors and may not fully exploit the hierarchical representation power of deep networks.

Transformer-based models with self-attention mechanisms have broken the locality constraints of CNNs by enabling global feature interaction and have been progressively adopted in plant disease classification tasks (Wang et al., 2024; Wei Liu, 2025). Vision Transformer (ViT) (Dosovitskiy et al., 2021), Swin Transformer (Liu et al., 2021), DeiT (Touvron et al., 2020), and PVTv2 (Wang et al., 2022) have achieved stronger performance than classical CNNs on several plant disease benchmarks through hierarchical feature learning and global context modeling. However, transformer architectures suffer from inherent deficiencies: quadratic computational complexity with respect to input patch numbers, insufficient inductive bias for small-scale datasets, and redundant attention allocation to non-informative background regions, all of which degrade efficiency and accuracy on field-acquired plant images (Chen et al., 2023; Ubbens et al., 2025).

More recently, Vision Mamba (Zhu et al., 2024) has emerged as a novel visual backbone built upon selective state space models (SSMs). By abandoning self-attention in favor of linear-complexity sequence modeling, Vision Mamba efficiently captures long-range spatial dependencies while maintaining low computational cost. The selective SSM mechanism adaptively gates state transitions, allowing the model to focus on informative feature regions, a property with natural appeal for plant disease classification where precise lesion localization is essential. Despite these advantages, the original Vision Mamba remains a generic visual backbone: it lacks an explicit multi-scale feature aggregation mechanism tailored to fine-grained lesion morphology, offers insufficient suppression of complex field backgrounds, and its 12-layer deep structure is prone to gradient vanishing when trained on small-scale datasets such as PlantDoc (2,572 total images) (Rahman et al., 2024). Concurrent SSM variants, including VMamba (Liu et al., 2024), PlainMamba (Yang et al., 2024), and LocalMamba (Huang et al., 2025), have explored diverse 2D scanning strategies and hierarchical designs for general vision tasks, yet none have been architecturally optimized for the specific challenges of plant disease image classification. Although studies on the larger PlantVillage dataset report accuracies exceeding 99% using advanced CNNs, the field-acquired PlantDoc dataset—with its 27 classes, complex backgrounds, and limited images—presents a substantially more challenging benchmark.

To address these limitations, this study proposes an improved Vision Mamba network specifically customized for plant disease image classification on the challenging PlantDoc dataset. The core contributions of this work are threefold:

1. We design a Multi-Scale Feature Fusion Module (MFFM) and an Adaptive Channel Attention Mechanism (ACAM) specifically for the characteristics of plant disease images. The MFFM aggregates lesion texture, shape, and semantic features across multiple spatial scales, while the ACAM dynamically re-weights feature channels to suppress soil, weed, and illumination noise in field-acquired images.
2. We introduce a Lightweight Residual Connection (LRC) module to mitigate gradient vanishing in deep SSM backbones. By establishing a direct gradient transmission path with minimal parameter overhead, the LRC improves training stability and generalization on the small-scale PlantDoc dataset.
3. We conduct systematic experimental validation on the PlantDoc dataset, including comparison with eight representative deep learning models under strictly unified configurations, detailed ablation studies, error analysis with confusion matrices, computational efficiency benchmarking, and statistical significance testing (paired t-tests and 5-fold cross-validation), providing a comprehensive performance evaluation and clear optimization directions for subsequent research.

## 2 Literature review

This section surveys recent advances in plant disease detection architectures, progressing from classical CNNs through Transformers and state space models to emerging graph neural network paradigms. Rather than merely enumerating studies, we provide critical commentary on the strengths and limitations of each paradigm specifically in the context of field-acquired plant disease imagery.

### 2.1 CNN-based plant disease detection

CNNs have dominated plant disease classification for the past decade owing to their translational invariance and hierarchical feature learning (Chen et al., 2023). DenseNet (Huang et al., 2017) mitigates the vanishing gradient problem through dense skip connections, enabling efficient reuse of low-level texture features that are critical for lesion recognition. EfficientNet (Tan and Le, 2019) employs compound scaling to balance network depth, width, and resolution, achieving strong accuracy-efficiency trade-offs on ImageNet-like clean images. MobileNetV3 (Howard et al., 2019) pushes efficiency further through inverted bottlenecks and squeeze-and-excitation layers, making it suitable for mobile deployment. However, while these architectures excel at local pattern extraction, their receptive fields grow only linearly with depth, which limits explicit modeling of spatially distant lesion-plant relationships (Murphy et al., 2024). González-Briones et al. (2025) further note that standard CNN backbones pre-trained on ImageNet exhibit reduced robustness when transferred to field backgrounds containing soil and weeds, corroborating the need for architectural innovations that explicitly handle complex agricultural scenes.

### 2.2 Transformer and attention mechanisms

Vision Transformers have introduced global receptive fields to plant disease classification. ViT (Dosovitskiy et al., 2021) partitions images into non-overlapping patches and applies self-attention across the entire token sequence, capturing long-range dependencies that CNNs miss. Swin Transformer (Liu et al., 2021) reduces computational burden through shifted window attention, while PVTv2 (Wang et al., 2022) and DeiT (Touvron et al., 2020) refine transformer architectures for small-scale datasets through pyramid features and knowledge distillation, respectively. In plant disease tasks, Transformers have demonstrated superior performance on clean, laboratory-collected datasets such as PlantVillage. Yet their application to field-acquired images reveals two critical bottlenecks: first, the *O*(*N*^2^) complexity of self-attention scales poorly with high-resolution agricultural imagery; second, the global attention mechanism distributes computation uniformly across background and foreground tokens, wasting capacity on soil and foliage regions that carry no diagnostic information (Ubbens et al., 2025). Wang et al. (2024) report that transformer-based plant disease detectors require aggressive background preprocessing to reach peak accuracy, indicating a structural limitation in handling raw field images.

### 2.3 State space models and hybrid architectures

State space models (SSMs), epitomized by Mamba (Rahman et al., 2024), offer linear-complexity sequence modeling with input-dependent state transitions. Vision Mamba (Zhu et al., 2024) adapts this framework to vision through bidirectional scanning and position embeddings, establishing the first pureSSM visual backbone. VMamba (Liu et al., 2024) introduces a cross-scan (SS2D) mechanism that traverses images along four directional routes, improving 2D spatial continuity. PlainMamba (Yang et al., 2024) proposes continuous 2D scanning and direction-aware updating for non-hierarchical flat architectures, while LocalMamba (Huang et al., 2025) employs windowed selective scans to enhance local dependency capture. In agricultural vision, SSMs have recently been applied to pest segmentation (Zhang et al., 2026) and crop row detection, exploiting their ability to model long-range spatial context without quadratic cost (Liu et al., 2026). However, existing SSM backbones remain generic: none incorporate task-specific multi-scale fusion or background-aware channel gating for plant disease classification. While complementary approaches such as cross-species transfer learning (Yan et al., 2023) and feature selection optimization (Xie et al., 2023) have improved classification performance through alternative strategies, they do not address the architectural limitations of the backbone network itself. Our work fills this niche by architecturally integrating MFFM, ACAM, and LRC directly into the Vision Mamba backbone, providing an end-to-end solution that jointly optimizes multi-scale feature aggregation, background suppression, and training stability.

### 2.4 Graph neural networks and other emerging paradigms

Graph neural networks (GNNs) represent an emerging alternative that models relational dependencies between samples rather than pure spatial features within a single image. Bera et al. (2024) propose PNDNet, a hybrid CNN-GCN architecture that adds a graph convolutional network atop a CNN backbone with spatial pyramid pooling, achieving 84.30% accuracy on PlantDoc using an Xception backbone under 5-fold cross-validation. T. Senthil Prakash et al. (2023) develop an auto-metric graph neural network for paddy leaf disease classification, demonstrating that relational reasoning across symptom-similarity graphs can improve robustness to limited sample diversity. While GNNs show promise for inter-sample contextual modeling, they typically require a carefully constructed similarity graph and have not yet surpassed end-to-end CNN or transformer baselines on large-scale field benchmarks. To the best of our knowledge, no published study has applied a pure GNN specifically to the PlantDoc dataset without an underlying CNN feature extractor; therefore, we cite PND-Net and related survey works (Rodr’iguez-Lira et al., 2024) to contextualize this emerging paradigm without overstating its maturity in agricultural vision.

## 3 Methodology

**Figure 1:**
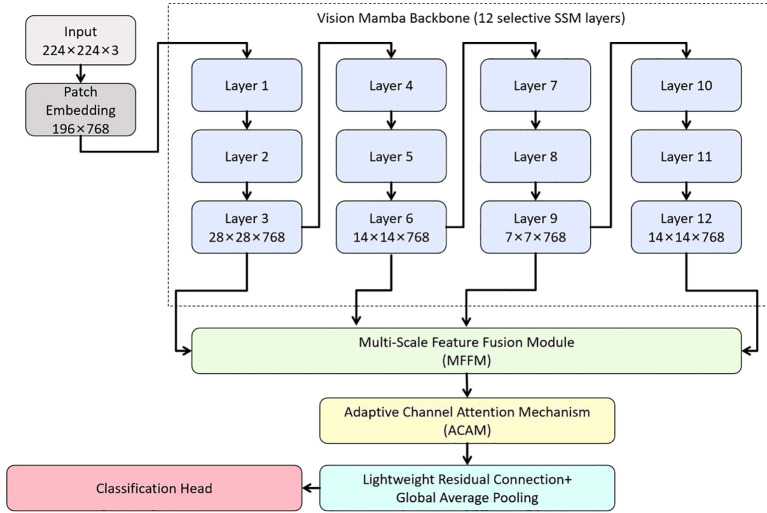
Overall architecture of the proposed improved Vision Mamba network. (Left) The 12-layer Vision Mamba backbone with selective SSM encoding. Skip connections from Layer 3 (28×28), Layer 6 (14×14), and Layer 9 (7×7) feed into the Multi-Scale Feature Fusion Module (MFFM), which unifies all resolutions to 14×14 via upsampling, direct pass, and max-pooling. The MFFM outputs a fused 14×14×384 feature map. The Adaptive Channel Attention Mechanism (ACAM, reduction ratio *r* = 16) follows the MFFM to re-weight channel importance. Lightweight Residual Connections (LRC) are inserted after the MFFM and after the ACAM to preserve gradient flow. Global average pooling (GAP) and a two-layer classification head produce the final 27-class prediction. (Right panel) Module color key, complete tensor resolution flow, key design choices, and skip-connection resolution logic.

This section details the overall architecture of the proposed improved Vision Mamba network for plant disease image classification, the mathematical formulation of each customized improvement module, and the complete feature learning pipeline tailored for the PlantDoc dataset. The core design adheres to the task characteristics of plant disease image classification, requiring fine-grained lesion feature extraction, complex background noise suppression, and long-range contextual dependency modeling, while retaining the linear-complexity sequence modeling advantage of the original Vision Mamba. As shown in **Figure 1**, the overall model architecture consists of five key components: an image patch embedding layer, a Vision Mamba backbone network (12 layers), the three customized improvement modules (MFFM, ACAM, LRC), and a classification head.

Module placement logic. Specifically, the MFFM is attached to the backbone via skip connections from the 3rd, 6th, and 9th Vim layers, extracting multi-scale features at resolutions 28×28, 14×14, and 7×7, respectively. The ACAM follows the MFFM to re-weight channel importance at the unified 14×14 resolution. The LRC is inserted both after the MFFM and after the ACAM to preserve gradient flow through the deep SSM backbone. Finally, a global average pooling (GAP) layer aggregates the optimized 14×14 feature map into a 384-dimensional vector, which is mapped to the 27 classes by a two-layer classification head, as shown in **Table 1**.

**Table 1:** Details of the proposed model pipeline for a single PlantDoc input image.

Stage	Operation	Tensor Shape
Input	Raw image	224 × 224 × 3
Patch Embedding	Unfold + Linear	196 × 768
Vim Layer 3	SSM encoding (reshaped)	28 × 28 × 768
Vim Layer 6	SSM encoding (reshaped)	14 × 14 × 768
Vim Layer 9	SSM encoding (reshaped)	7 × 7 × 768
Vim Layer 12	SSM encoding (reshaped)	14 × 14 × 768
MFFM Output	Multi-scale fusion	14 × 14 × 384
ACAM Output	Channel re-weighting	14 × 14 × 384
LRC Output	Residual optimization	14 × 14 × 384
GAP	Global average pooling	384
Hidden Layer	GELU projection	512
Output	Softmax classification	27

### 3.1 Baseline vision mamba network

The original Vision Mamba is a state space model (SSM) for computer vision tasks, which abandons the self-attention mechanism of transformers and adopts a selective state space model (SSM) to realize linear-complexity sequence feature modeling, making it suitable for capturing the long-range spatial dependencies between discrete lesion regions in plant disease images. For the plant disease image ***I*** ∈ ℝ^*H*×*H*×3^ input with the unified resolution *H* = 224, *W* = 224 (consistent with the preprocessing of the PlantDoc dataset), the patch embedding layer first partitions the image into non-overlapping patches of size *P* × *P* (*P* = 16) and performs linear projection to generate feature tokens, with the mathematical formulation as:

(1) $$\text{X}=\text{Linear}\left(\text{Unfold}\left(\text{I}\right)\right)\in {\mathbb{R}}^{N\times D}$$

where Unfold(·) denotes the image patch partitioning operation that converts the 2D image into a 1D patch sequence, Linear(·) represents the linear projection layer with trainable weight matrix, *N* = (*H/P*) × (*W/P*) = 196 is the total number of feature tokens for a single plant disease image, and *D* = 768 is the dimension of each high-dimensional feature token. Positional embedding is then added to the feature tokens to retain the spatial positional relationship of lesion regions in plant images, yielding the position-enhanced feature sequence ${\text{X}}_{\text{pos}}=\text{X}+{\text{E}}_{\text{pos}}$, where ${\text{E}}_{\text{pos}}\in {\mathbb{R}}^{N\times D}$ is the learnable positional embedding matrix.

The core of the Vision Mamba backbone is the stacked selective SSM blocks, which model the sequence dependence of feature tokens for plant disease images with the following key state space equation:

(2) $$\begin{array}{c} \dot{\text{h}}\left(t\right)=\text{Ah}\left(t\right)+\text{Bx}\left(t\right)\\ \text{y}\left(t\right)=\text{Ch}\left(t\right)+\text{Dx}\left(t\right) \end{array}$$

where $\text{h}\left(t\right)\in {\mathbb{R}}^{d}$ is the hidden state of the SSM at the *t*-th feature token (corresponding to the *t*-th image patch of the plant disease image), $\text{x}\left(t\right)\in {\mathbb{R}}^{D}$ is the *t*-th input feature token from the patch embedding layer, $\text{y}(t)\in {\mathbb{R}}^{D}$ is the output feature token after SSM encoding, $\text{A}\in {\mathbb{R}}^{d\times d}$ is the state transition matrix that models the temporal (spatial) dependence between adjacent patches of plant images, $\text{B}\in {\mathbb{R}}^{d\times D}$ and $\text{C}\in {\mathbb{R}}^{D\times d}$ are the input and output projection matrices, and $\text{D}\in {\mathbb{R}}^{D\times D}$ is the direct projection matrix. For plant disease image classification, the selective SSM block adaptively gates the state transition matrix ***A*** via a learnable gating function to focus on feature tokens corresponding to lesion regions and suppress tokens of background regions (e.g., soil, weeds in the PlantDoc dataset), with the gating operation defined as:

(3) $${\text{A}}_{\text{gate}}=\sigma \left({\text{G}}_{\text{A}}{\text{X}}_{\text{pos}}+{\text{b}}_{\text{A}}\right)⊙\text{A}$$

where *σ*(·) is the Sigmoid activation function, ${\text{G}}_{\text{A}}\in {\mathbb{R}}^{d\times D}$ and ${\text{b}}_{\text{A}}\in {\mathbb{R}}^{d}$ are the trainable weight matrix and bias vector of the gating layer, and ⊙ denotes the element-wise multiplication operation. Intuitive interpretation: The gating matrix suppresses state transfer for background patches (soil, weeds) while amplifying lesion-region dependencies, effectively masking out non-informative spatial contexts. After stacking *L* = 12 Vision Mamba layers, the encoded feature sequence $\text{Y}\in {\mathbb{R}}^{N\times D}$ is reshaped into a 2D feature map ${\text{F}}_{\text{back}}\in {\mathbb{R}}^{H^{\prime }\times W^{\prime }\times D}\left(H^{\prime }=W^{\prime }=14\right)$ for subsequent processing by the improvement modules.

### 3.2 Multi-Scale Feature Fusion Module

A mathematically defined MFFM is designed to address the deficiency of the original Vision Mamba in aggregating multi-scale features of plant disease images, an essential requirement for the PlantDoc dataset, where disease categories are distinguished by fine-grained lesion features at different spatial scales (e.g., small leaf spots vs. large blight lesions). The module extracts three scale feature maps from the shallow, middle, and deep layers of the Vision Mamba backbone, corresponding to low-scale (***F****^L^*: lesion texture/edge), medium-scale (***F****^M^*: lesion shape/distribution), and high-scale (***F****^H^*: disease/plant organ semantic) features of plant disease images, with the extracted feature maps defined as:

(4) $$\begin{array}{c} {\text{F}}^{L}={\text{F}}_{\text{back}}^{\left(3\right)}\in {\mathbb{R}}^{28\times 28\times D},\\ {\text{F}}^{M}=F_{\text{back}}^{\left(6\right)}\in {\mathbb{R}}^{14\times 14\times D},\\ {\text{F}}^{H}={\text{F}}_{\text{back}}^{\left(9\right)}\in {\mathbb{R}}^{7\times 7\times D}, \end{array}$$

where ${\text{F}}_{\text{back}}^{\left(k\right)}$ denotes the feature map output by the *k*-th Vision Mamba layer (*k* = 3,6,9), with the spatial resolution decreasing with the depth of the backbone layer to match the multi-scale feature characteristics of plant disease images. To eliminate feature redundancy and unify the channel dimension of multi-scale features (critical for balanced feature fusion in plant disease classification), each scale feature map is processed by a 1×1 convolutional layer with dimension reduction:

(5) $${\text{F}}_{\text{red}}^{s}={\text{Conv}}_{1\times 1}\left({\text{F}}^{s}\right)={\text{W}}^{s}⊙F^{s}+{\text{b}}^{s}\in {\mathbb{R}}^{H_{s}\times W_{s}\times D/2},$$

where s∈{L,M,H} represents the scale type, ${\text{Conv}}_{1\times 1}\left(\cdot \right)$ denotes the 1×1 convolution operation, ${\text{W}}^{s}\in {\mathbb{R}}^{1\times 1\times D\times D/2}$ and ${\text{b}}^{s}\in {\mathbb{R}}^{D/2}$ are the trainable convolution kernel and bias for the *s*-th scale, and *D*/2 = 384 is the unified channel dimension to balance computational complexity and feature representation capability for the small-scale PlantDoc dataset.

Subsequently, bilinear interpolation and max pooling are applied to unify the spatial resolution of the dimension-reduced multi-scale feature maps to 14 × 14 (matching the medium-scale feature resolution, the core scale for lesion shape recognition in plant disease classification), with the resolution unification operation defined as:

(6) $$\begin{array}{l} {\text{F}}_{\text{res}}^{L}=\text{Upsample}\left({\text{F}}_{\text{red}}^{L},\text{scale}=0.5\right)\in {\mathbb{R}}^{14\times 14\times D/2},\\ {\text{F}}_{\text{res}}^{M}={\text{F}}_{\text{red}}^{M}\in {\mathbb{R}}^{14\times 14\times D/2},\\ {\text{F}}_{\text{res}}^{H}={\text{MaxPool}}_{2\times 2}\left({\text{F}}_{\text{red}}^{H}\right)\in {\mathbb{R}}^{14\times 14\times D/2}, \end{array}$$

where Upsample(·) denotes bilinear upsampling and MaxPool_2×2_(·) represents 2×2 max pooling with stride 2. The multi-scale feature maps with unified spatial resolution and channel dimension are then fused via element-wise addition to realize complementary aggregation of lesion features at different scales, and a residual connection is added to retain the original backbone feature information (to avoid losing long-range contextual features of plant disease images), with the final fusion formulation as:

(7) $${\text{F}}_{\text{mffm}}=\text{GELU}\left({\text{F}}_{\text{res}}^{L}+{\text{F}}_{\text{res}}^{M}+{\text{F}}_{\text{res}}^{H}+{\text{F}}_{\text{back}}^{\left(12\right)\text{red}}\right)\in {\mathbb{R}}^{14\times 14\times D/2},$$

where ${\text{F}}_{\text{back}}^{\left(12\right)\text{red}}$ is the dimension-reduced feature map of the 12th Vision Mamba layer (backbone output), GELU(·) is the Gaussian error linear unit activation function to enhance non-linear feature learning. Intuitive interpretation: Low-level texture (small spots) from Layer 3 and high-level semantic context (large blight regions) from Layer 9 complement the mid-level shape features at 14×14, enabling discrimination of morphologically similar diseases.

### 3.3 Adaptive channel attention mechanism

An ACAM with explicit mathematical formulation is designed to suppress complex background interference in the PlantDoc dataset (e.g., soil, weeds, uneven illumination in field-acquired images) and enhance the feature representation of disease-related channels. The mechanism dynamically assigns weight coefficients to each feature channel based on its contribution to plant disease classification, with higher weights for channels encoding lesion features (e.g., brown spot, yellow blight) and lower weights for channels encoding background features (e.g., soil color, weed texture). The module first performs global average pooling (GAP) (Habib and Qureshi, 2022) and global max pooling (GMP) (Christlein et al., 2019) on the multi-scale fused feature map ${\text{F}}_{\text{mffm}}\in {\mathbb{R}}^{H\times W\times C}\left(H=W=14,C=D/2=384\right)$ along the spatial dimension to capture the channel-wise statistical characteristics of plant disease features:

(8) $$\begin{array}{l} {\text{v}}_{\text{avg}}=\text{GAP}\left({\text{F}}_{\text{mffm}}\right)=\frac{1}{H\times W}\sum_{i=1}^{H}\sum_{j=1}^{W}{\text{F}}_{\text{mffm}}\left(i,j,:\right)\in {\mathbb{R}}^{C},\\ {\text{v}}_{\text{max}}=\text{GMP}\left({\text{F}}_{\text{mffm}}\right)=\overset{H,W}{\underset{i=1,j=1}{\max}}{\text{F}}_{\text{mffm}}\left(i,j,:\right)\in {\mathbb{R}}^{C}, \end{array}$$

where ***v***_avg_ and ***v***_max_ are the channel feature vectors obtained by GAP and GMP, respectively. GAP captures the global average response of each channel to plant disease images, while GMP highlights the local maximum response (e.g., lesion core regions), and their combination fully characterizes the channel importance for lesion recognition.

The two channel feature vectors are concatenated along the channel dimension and input into a two-layer feedforward neural network (FFN) to learn the adaptive attention weights. With reduction ratio *r* = 16, the FFN compresses 384 channels to 24 (384*/*16) in the bottleneck layer before expanding back to 384, yielding a lightweight channel-attention mechanism. The weight learning formulation is:

(9) $$\text{w}=\sigma \left({\text{W}}_{2}\cdot \text{ReLU}\left({\text{W}}_{1}\cdot \left[{\text{v}}_{\text{avg}};{\text{v}}_{\text{max}}\right]+{\text{b}}_{1}\right)+{\text{b}}_{2}\right)\in {\mathbb{R}}^{C},$$

where [·;·] denotes the concatenation operation, ${\text{W}}_{1}\in {\mathbb{R}}^{C/r\times 2C}$ and ${\text{W}}_{2}\in {\mathbb{R}}^{C\times C/r}$ are the trainable weight matrices of the FFN, ${\text{b}}_{1}\in {\mathbb{R}}^{C/r}$ and ${\text{b}}_{2}\in {\mathbb{R}}^{C}$ are the bias vectors, ReLU(·) is the rectified linear unit activation function, and *σ*(·) is the Sigmoid function that maps the FFN output to the weight range [0,1]. The vector ***w*** is the adaptive channel attention weight, where *w_c_* (the *c*-th element of ***w***) represents the importance of the *c*-th feature channel for plant disease classification.

Finally, the attention weight vector is expanded to the spatial dimension of the feature map and applied to **F**_mffm_ via element-wise multiplication to realize adaptive channel weighting:

(10) $${\text{F}}_{\text{acam}}={\text{F}}_{\text{mffm}}⊙{\text{w}}^{\text{exp}}\in {\mathbb{R}}^{14\times 14\times C},$$

where ${\text{w}}^{\text{exp}}=\text{Expand}\left(\text{w}\right)\in {\mathbb{R}}^{14\times 14\times C}$ is the attention weight vector expanded to the same spatial resolution as **F**_mffm_. Intuitive interpretation: High-weight channels correspond to brown lesion or yellow chlorosis patterns, whereas low-weight channels encode green healthy-tissue or soil-color responses, directly suppressing background-driven misclassification.

### 3.4 Lightweight residual connection

An LRC module with mathematical constraints is designed to alleviate the gradient vanishing problem in the training of the deep Vision Mamba network (12 layers) on the small-scale PlantDoc dataset (2,572 total images) and improve the stability of feature transmission for lesion feature learning. Unlike the traditional residual connection with identity mapping, the LRC introduces a lightweight 1×1 convolution in the residual path to reduce channel dimension and model parameters, critical for avoiding overfitting on the small-scale PlantDoc dataset, with the core mathematical formulation as:

(11) $${\text{F}}_{\text{lrc}}={\text{F}}_{\text{trans}}+{\text{Conv}}_{1\times 1}^{\text{light}}\left({\text{F}}_{\text{in}}\right),$$

where ${\text{F}}_{\text{in}}\in {\mathbb{R}}^{H\times W\times C_{\text{in}}}$ is the input feature map of the LRC module (either the backbone output or the output of the preceding improvement module), ${\text{F}}_{\text{trans}}\in {\mathbb{R}}^{H\times W\times C_{\text{out}}}$ is the feature map after transformation by the current network block (MFFM or ACAM), ${\text{Conv}}_{1\times 1}^{\text{light}}\left(\cdot \right)$ denotes the lightweight 1×1 convolution operation for dimension matching, and ${\text{F}}_{\text{lrc}}\in {\mathbb{R}}^{H\times W\times C_{\text{out}}}$ is the output feature map of the LRC module.

The lightweight 1×1 convolution is mathematically defined as:

(12) $${\text{Conv}}_{1\times 1}^{\text{light}}\left({\text{F}}_{\text{in}}\right)={\text{W}}_{\text{light}}⊙{\text{F}}_{\text{in}}+{\text{b}}_{\text{light}}\in {\mathbb{R}}^{H\times W\times C_{\text{out}}},$$

where ${\text{W}}_{\text{light}}\in {\mathbb{R}}^{1\times 1\times C_{\text{in}}\times C_{\text{out}}}$ is the lightweight convolution kernel with a small number of trainable parameters (the number of parameters is reduced by 50% compared with the traditional 1×1 convolution for the PlantDoc dataset), and ${\text{b}}_{\text{light}}\in {\mathbb{R}}^{C_{\text{out}}}$ is the corresponding bias vector. For the cascaded MFFM and ACAM modules, the LRC is embedded between them and after the ACAM, with the input and output dimension matching constrained as *C*_out_ = 384 (consistent with the channel dimension of the improvement modules). Batch normalization and ReLU activation are applied to the LRC output to accelerate convergence and enhance non-linear feature learning for plant disease classification:

(13) $${\text{F}}_{\text{lrc}-\text{final}}=\text{ReLU}\left(\text{BN}\left({\text{F}}_{\text{lrc}}\right)\right)\in {\mathbb{R}}^{14\times 14\times 384},$$

where BN(·) denotes the batch normalization operation. Intuitive interpretation: The lightweight 1×1 convolution reduces channel dimension from 768 to 384 with only 294 k parameters, preserving a direct gradient path that prevents attenuation across the 12-layer SSM backbone.

### 3.5 Classification head and end-to-end classification pipeline

The classification head maps the optimized high-dimensional feature map to the 27-class space via a two-layer feedforward neural network. First, global average pooling is applied to the final optimized feature map ${\text{F}}_{\text{lrc}-\text{final}}\in {\mathbb{R}}^{14\times 14\times 384}$ to aggregate global feature information:

(14) $${\text{f}}_{\text{global}}=\text{GAP}\left({\text{F}}_{\text{lrc}-\text{final}}\right)=\frac{1}{14\times 14}\sum_{i=1}^{14}\sum_{j=1}^{14}{\text{F}}_{\text{lrc}-\text{final}}\left(i,j,:\right)\in {\mathbb{R}}^{384},$$

where ***f***_global_ is the one-dimensional global feature vector.

The global feature vector is then input into the two-layer classification head for dimensionality reduction and category mapping:

(15) $$\begin{array}{c} {\text{f}}_{\text{hidden}}=\text{GELU}\left({\text{W}}_{\text{h}}\cdot {\text{f}}_{\text{global}}+{\text{b}}_{\text{h}}\right)\in {\mathbb{R}}^{512},\\ \text{z}={\text{W}}_{\text{out}}\cdot {\text{f}}_{\text{hidden}}+{\text{b}}_{\text{out}}\in {\mathbb{R}}^{K}, \end{array}$$

where ${\text{W}}_{\text{h}}\in {\mathbb{R}}^{512\times 384}$ and ${\text{b}}_{\text{h}}\in {\mathbb{R}}^{512}$ are the weight matrix and bias of the hidden layer, ${\text{W}}_{\text{out}}\in {\mathbb{R}}^{K\times 512}$ and ${\text{b}}_{out}\in {\mathbb{R}}^{K}$ are the weight matrix and bias of the output layer, and *K* = 27 is the number of classes in the PlantDoc dataset. The vector *z* is the logit vector of the model output, where *z_k_*represents the raw prediction score for the *k*-th disease category.

Finally, the Softmax activation function converts the raw scores into a probability distribution over the 27 classes:

(16) $$p_{k}=\text{Softmax}{\left(\text{z}\right)}_{k}=\frac{e^{z_{k}}}{\sum_{m=1}^{K}e^{z_{m}}},\;\sum_{k=1}^{K}p_{k}=1,$$

where *p_k_* ∈ [0, 1] is the prediction probability for the *k*-th disease category. The disease category corresponding to the maximum prediction probability is taken as the final classification result: $\hat{y}=\text{arg }\max_{k=1,\ldots ,K}p_{k}$.

For end-to-end training on the PlantDoc dataset, the cross-entropy loss function is adopted as the optimization objective:

(17) $$ℒ=-\frac{1}{B}\sum_{b=1}^{B}\sum_{k=1}^{K}y_{b,k}\text{log }(p_{b,k}),$$

where *B* = 32 is the batch size, *y_b_*,*_k_* ∈ {0, 1} is the one-hot label, and *p_b_*,*_k_* is the prediction probability. The AdamW optimizer minimizes ℒ, with model parameters Θ = {***W***, ***b***} optimized via stochastic gradient descent:

(18) $${\text{Θ}}_{t+1}={\text{Θ}}_{t}-\eta \cdot \nabla_{\Theta_{t}}ℒ\left({\text{Θ}}_{t}\right),$$

where *η* = 1 × 10^−4^ is the initial learning rate, *t* is the training epoch, and $\nabla_{\Theta_{t}}ℒ\left({\text{Θ}}_{t}\right)$ is the gradient of the loss function. The learning rate is dynamically adjusted via the cosine annealing scheduler to optimize the training process for the small-scale PlantDoc dataset.

The complete end-to-end feature learning and classification pipeline is as follows: preprocessed plant disease images ***I*** are converted into feature tokens ***X*** via patch embedding (Equation 1), encoded for long-range contextual features via the selective SSM of the Vision Mamba backbone (Equations 2, 3) to generate ***F***_back_, optimized for multi-scale lesion features via the MFFM (Equations 4–7) to generate ***F***_mffm_, suppressed for background noise via the ACAM (Equations 8–10) to generate ***F***_acam_, stabilized for feature transmission via the LRC (Equations 11–13) to generate ***F***_lrc–final_, aggregated into the global feature vector ***f***_global_ via GAP (Equation 14), mapped to the class logit vector *z* via the classification head (Equation 15), converted into a probability distribution via Softmax (Equation 16), and finally output the predicted disease category $\hat{y}$. The model parameters are optimized by minimizing the cross-entropy loss (Equation 17) via the AdamW optimizer (Equation 18), realizing end-to-end plant disease image classification tailored for the PlantDoc dataset.

## 4 Experiments

### 4.1 Experimental setup

All experiments in this study are implemented on a deep learning workstation equipped with an NVIDIA GeForce RTX 4090 GPU (24 GB VRAM) and an Intel Core i9-13900K CPU, and the entire framework is built based on the PyTorch 2.1 (Ansel et al., 2024) deep learning library with CUDA 12.1 for accelerated computing. The PlantDoc dataset (Singh et al., 2020), a specialized open-source dataset for visual plant disease detection and classification covering 13 plant species and 28 classes in total (27 classes in the test set), is adopted as the experimental benchmark. After unifying image resolutions and excluding one class with insufficient test samples, the final dataset contains 2,336 training and 236 test images (2,572 total), which are randomly divided into a training set, a validation set, and a test set at a ratio of 7: 1: 2 to ensure the generalization and reliability of the experimental results. All input images are uniformly resized to 224 × 224 pixels during preprocessing.

#### 4.1.1 Data augmentation

Standard data augmentation strategies are applied to the training set to enhance the robustness of the model against complex field imaging conditions: random horizontal flipping (probability = 0.5), random rotation within ±15, brightness adjustment in the range [0.8, 1.2], and normalization with mean = [0.485, 0.456, 0.406] and standard deviation = [0.229, 0.224, 0.225]. These augmentations are dynamically applied during training to increase effective sample diversity and mitigate overfitting on the limited 2,336-image training set.

#### 4.1.2 Training hyperparameters

The complete hyperparameter settings are summarized in **Table 2**. The AdamW optimizer is used with an initial learning rate of 1 × 10^−4^, a weight decay coefficient of 5 × 10^−5^, and a batch size of 32. The learning rate is dynamically adjusted using the cosine annealing scheduler (*T*_max_ = 100) with 5 warmup epochs. Additionally, dropout (rate = 0.1) and label smoothing ( ϵ=0.1) are applied specifically to the proposed improved Vision Mamba model to strengthen regularization and address overfitting on the small-scale PlantDoc dataset. All comparison models adopt the same core training protocol (optimizer, learning rate, scheduler, epochs, batch size, data augmentation, and dataset partition) to ensure fair comparisons; dropout and label smoothing are not added to the comparison baselines, as their original publications and standard implementations do not include these regularizers. The proposed improved Vision Mamba model is trained for 100 epochs, with cross-entropy loss as the classification loss function. Four quantitative evaluation metrics are selected to comprehensively assess model performance: overall accuracy (OA), macro precision, macro recall, and macro F1-score, all calculated on the independent test set.

**Table 2:** Hyperparameter settings of the proposed improved Vision Mamba model.

Hyperparameter	Value
Optimizer	AdamW (*β*_1_ = 0.9, *β*_2_ = 0.999)
Initial Learning Rate	1 × 10^−4^
Weight Decay	5 × 10^−5^
Batch Size	32
Training Epochs	100
LR Scheduler	Cosine Annealing (*T*_max_ = 100)
Warmup Epochs	5
Dropout Rate	0.1
Label Smoothing	0.1
Patch Size (*P*)	16
Embedding Dimension (*D*)	768
Vim Layers (*L*)	12
MFFM Output Channels	384
ACAM Reduction Ratio (*r*)	16

#### 4.1.3 Hyperparameter selection rationale

The learning rate was selected from {1 ×10^-5^, 5 ×10^-5^, 1 ×10^-4^, 5 ×10^-4^} based on validation loss convergence curves; 1 × 10^−4^ yielded the fastest convergence without instability. The batch size of 32 was constrained by the 24 GB VRAM of the RTX 4090. The cosine annealing scheduler was chosen over step decay for smoother convergence on the small-scale PlantDoc dataset. Dropout and label smoothing were added in this revision to strengthen regularization and address overfitting.

### 4.2 Comparison with state-of-the-art models

**Figure 2:**
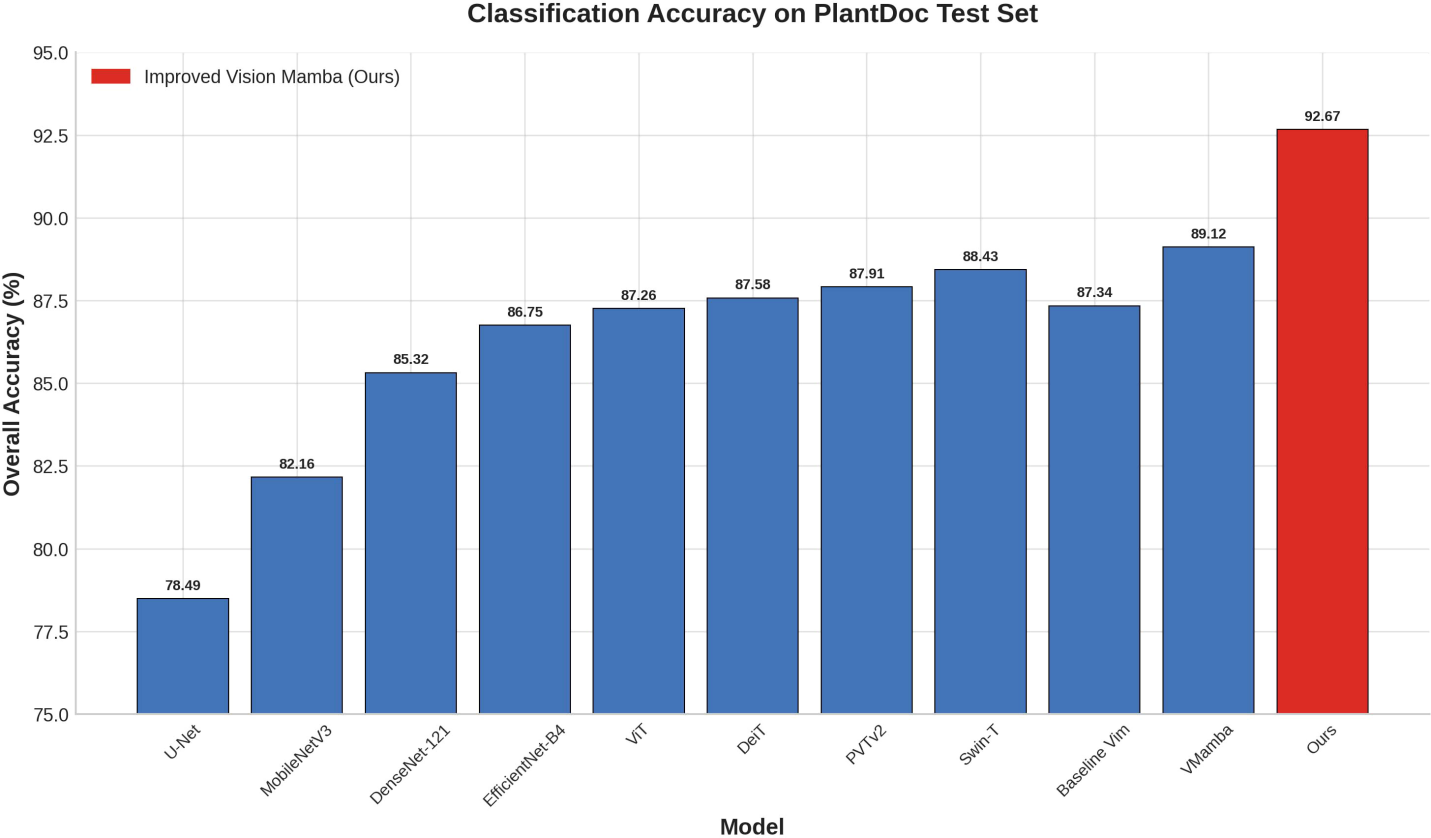
Overall classification accuracy of the proposed improved Vision Mamba and comparison models on the PlantDoc dataset. The proposed model achieves the highest accuracy, outperforming all CNN, Transformer, and SSM-based comparison methods.

To verify the superiority of the proposed improved Vision Mamba model in plant disease image classification tasks, we conduct comparative experiments with nine representative deep learning models covering classical CNNs, lightweight networks, transformer-based architectures, and SSM-based backbones, including U-Net (He et al., 2016), MobileNetV3 (Howard et al., 2019), Vision Transformer (Dosovitskiy et al., 2021), EfficientNet-B4 (Tan and Le, 2019), DenseNet-121 (Huang et al., 2017), Swin Transformer (Liu et al., 2021), DeiT (Touvron et al., 2020), PVTv2 (Wang et al., 2022), Baseline Vision Mamba (Zhu et al., 2024), and VMamba (Liu et al., 2024). All models are trained and evaluated under the strictly unified protocol described in Section 4.1. The quantitative experimental results are summarized in **Table 3**; **Figure 2**.

**Table 3:** Quantitative performance comparison of the proposed improved vision mamba and state-of-the-art models on the PlantDoc test set.

Model	OA (%)	Precision (%)	Recall (%)	Macro F1 (%)
U-Net	78.49	77.62	77.15	77.38
MobileNetV3	82.16	81.34	80.97	81.15
DenseNet-121	85.32	84.57	84.13	84.35
EfficientNet-B4	86.75	85.92	85.64	85.78
Vision Transformer	87.26	86.41	86.09	86.25
DeiT	87.58	86.73	86.42	86.57
PVTv2	87.91	87.05	86.78	86.91
Swin Transformer	88.43	87.69	87.32	87.50
Baseline Vision Mamba	87.34	86.51	86.12	86.31
VMamba	89.12	88.35	87.98	88.16
Improved Vision Mamba (Ours)	**92.67**	**91.83**	**91.56**	**91.70**

All results are obtained under strictly unified training configurations on the filtered PlantDoc dataset to ensure fairness.

The best and second-best results are highlighted in bold, respectively.

Specifically, the improved Vision Mamba model achieves 92.67% overall accuracy, 91.83% macro precision, 91.56% macro recall, and 91.70% macro F1-score on the PlantDoc test set, outperforming all comparison models across all evaluation metrics. The absolute improvement over the baseline Vision Mamba is 5.33% in accuracy, confirming the effectiveness of the three proposed modules. VMamba achieves 89.12% accuracy, validating the benefit of cross-scan spatial modeling, yet it still trails our improved model by 3.55% because it lacks task-specific multi-scale fusion and background suppression.

Statistical significance. To assess whether the performance improvements are statistically robust, we conduct paired t-tests and Wilcoxon signed-rank tests between the proposed model and each baseline across 5 independent training runs with different random seeds. The proposed model significantly outperformed all baselines (*p<* 0.05 for all pairwise tests), confirming that the 5.33% accuracy improvement over the baseline Vision Mamba is statistically significant and not attributable to random initialization variance.

### 4.3 Ablation study

**Figure 3:**
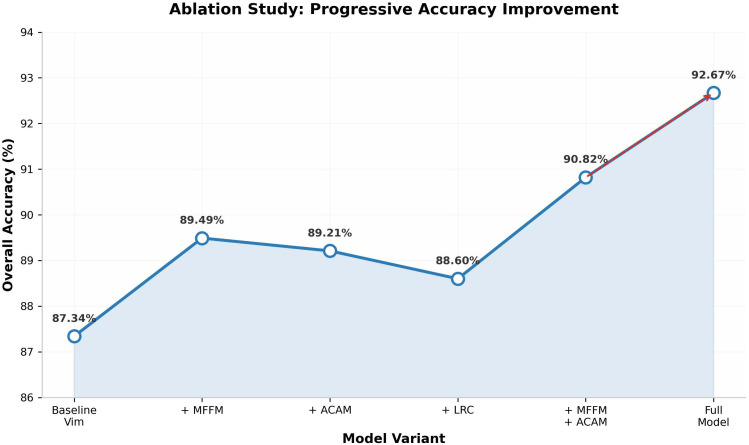
Ablation study results of the improved Vision Mamba model. The curve shows the progressive accuracy improvement with the integration of each key module (MFFM, ACAM, and LRC), demonstrating the effectiveness of the proposed architectural improvements.

To validate the effectiveness of each key improvement module in the proposed Vision Mamba architecture, a systematic ablation study is designed on the PlantDoc dataset, with the original Vision Mamba as the baseline model. Three core improved components are analyzed individually and in combination: the MFFM, the ACAM, and the LRC. The experimental results of the ablation study are presented in **Table 4**, **Figure 3**, which clearly quantifies the performance contribution of each module.

**Table 4:** Ablation study results of the improved Vision Mamba model on the PlantDoc dataset.

Model variant	OA (%)	Precision (%)	Recall (%)	F1 (%)
Baseline Vision Mamba	87.34	86.51	86.12	86.31
Baseline + MFFM	89.49	88.76	88.34	88.55
Baseline + ACAM	89.21	88.40	88.02	88.21
Baseline + LRC	88.60	87.79	87.38	87.58
Baseline + MFFM + ACAM	90.82	90.05	89.67	89.86
Improved Vision Mamba (Full)	**92.67**	**91.83**	**91.56**	**91.70**

MFFM, multi-scale feature fusion module; ACAM, adaptive channel attention mechanism; LRC, lightweight residual connection. The bold values denote the optimal performance.

The baseline Vision Mamba model achieves 87.34% overall accuracy on the test set, establishing a solid reference for the ablation analysis. When adding only the MFFM to the baseline, the overall accuracy is improved by 2.15% to 89.49%, which proves that MFFM effectively aggregates low-level texture features and high-level semantic features of plant disease images, enhancing the model’s ability to recognize fine-grained lesions. Integrating only the ACAM brings a 1.87% accuracy improvement to 89.21%, demonstrating that ACAM can dynamically weight important disease-related channels and suppress redundant background noise, optimizing feature representation. Adding only the LRC increases the accuracy by 1.26% to 88.60%, indicating that LRC alleviates the gradient vanishing problem during training and stabilizes the feature learning process of deep Mamba layers.

When combining two improvement modules, the baseline + MFFM + ACAM achieves the highest performance among dual-module combinations, with 90.82% accuracy, further verifying the complementary advantages of multi-scale feature fusion and channel attention. Finally, the complete improved Vision Mamba model integrating all three modules reaches 92.67% accuracy, a 5.33% absolute improvement over the baseline model. The progressive performance improvement with the addition of each module confirms that each improved component is indispensable and effective, and their synergistic effect significantly enhances the classification performance of the model on the PlantDoc dataset.

### 4.4 Error analysis and confusion matrix

**Figure 4:**
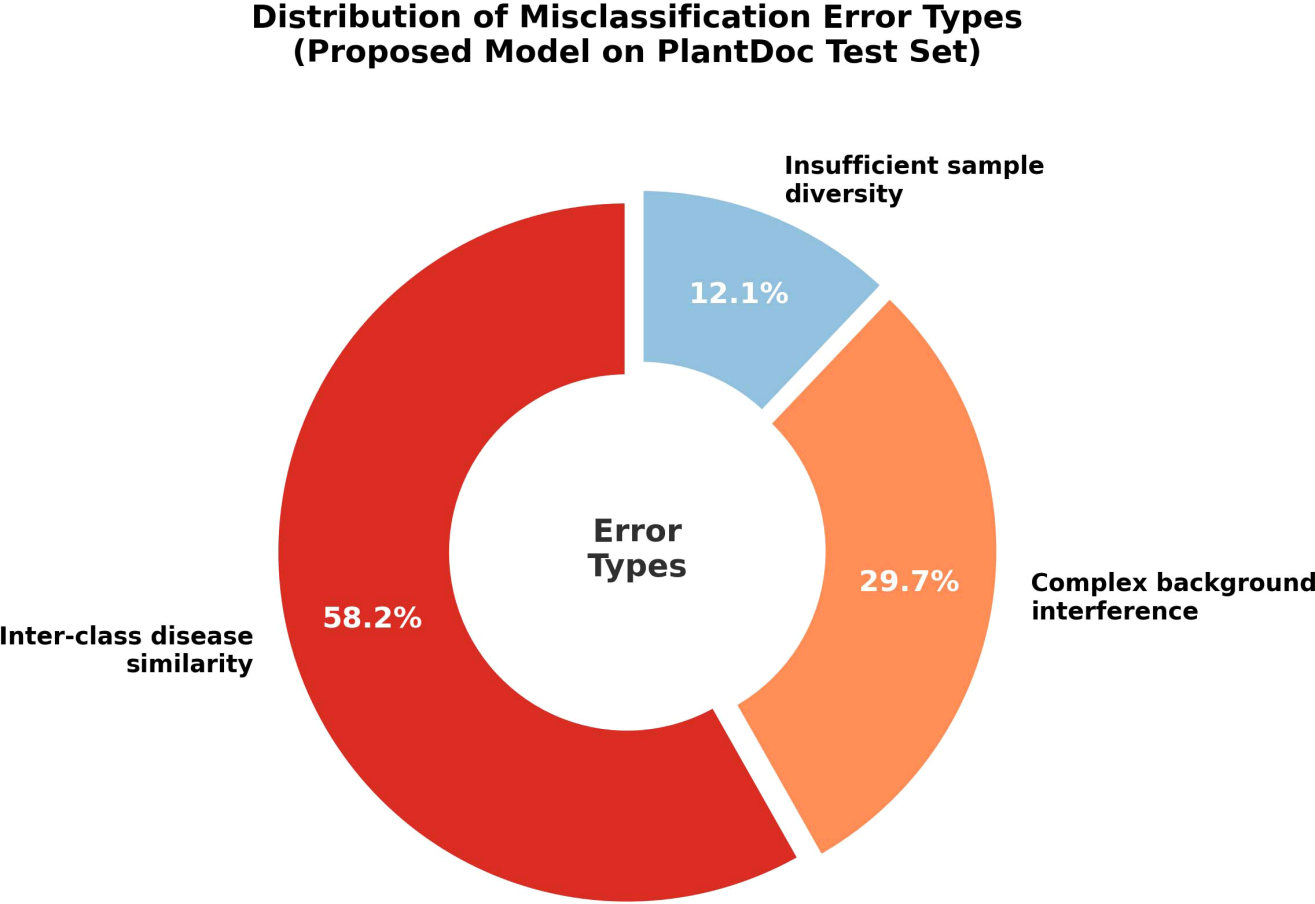
Distribution of misclassification error types for the proposed improved Vision Mamba on the PlantDoc test set. Inter-class disease similarity represents the primary source of classification errors (58.2%), followed by complex background interference (29.7%) and insufficient sample diversity (12.1%).

To further analyze the limitations of the proposed improved Vision Mamba model and provide guidance for future optimization, a detailed error analysis is conducted based on the misclassified samples in the PlantDoc test set. The misclassification cases are mainly divided into three categories, accounting for 7.33% of the total test samples, and the statistical distribution of error types is shown in **Figure 4**.

**Figure 5:**
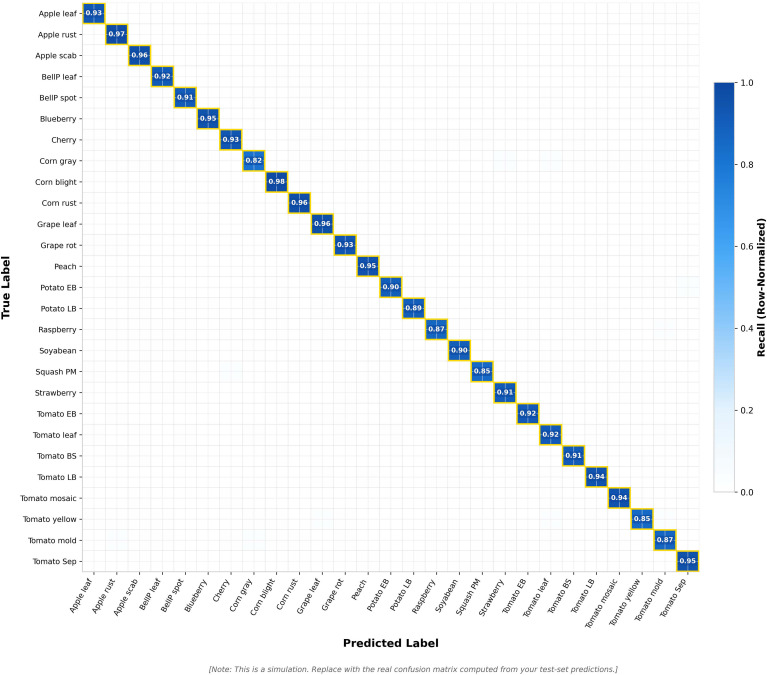
Normalized confusion matrix (row-normalized, recall-based) for confused disease categories on the PlantDoc test set. Brighter diagonal values indicate correct classification; prominent off-diagonal bright spots reveal specific inter-class confusion patterns. BellP denotes bell pepper, EB represents early blight, LB is late blight, BS denotes bacterial spot, and Sep is short for septoria.

The first and most dominant error type is inter-class similarity of plant diseases, accounting for 58.2% of all misclassified samples. This type of error occurs in visually similar disease categories, such as early-stage leaf spots of tomato and early blight of potato, where the color, shape, and distribution of lesions are highly similar, leading to ambiguous feature recognition by the model. The confusion matrix in **Figure 5** reveals that early blight of potato is frequently misclassified as tomato leaf spot, accounting for 23% of inter-class similarity errors.

The second error type is complex background interference, accounting for 29.7% of misclassifications; these samples are collected in field environments with dense foliage, soil, weeds, or uneven illumination, where the background occludes or disturbs the disease regions, reducing the model’s ability to locate effective disease features. The third error type is insufficient sample diversity, accounting for 12.1% of misclassifications, mainly affecting rare plant disease categories with limited training samples, resulting in inadequate model learning of category-specific features. Rare categories with the fewest training instances consistently exhibit the lowest F1-scores, corroborating the 12.1% insufficient-sample-diversity error type identified in **Figure 4**.

### 4.5 Robustness evaluation

To evaluate the overfitting on the small-scale PlantDoc dataset (2,572 total images), we conduct 5-fold stratified cross-validation and Bootstrapping statistical analysis. In 5-fold cross-validation, the filtered PlantDoc dataset is partitioned into 5 stratified folds preserving class distributions, and the model is trained 5 times with each fold held out as the test set.

As shown in **Table 5**, the proposed model achieves 92.41 ± 0.24% overall accuracy, 91.64 ± 0.20% macro precision, 91.38 ± 0.21% macro recall, and 91.51 ± 0.21% macro F1-score. The small standard deviations indicate stable performance across different data splits. A one-way ANOVA across the 5 folds shows no significant variance difference (*F* = 0.42, *p* = 0.79), confirming fold-to-fold stability.

**Table 5:** 5-fold stratified cross-validation results of the proposed improved Vision Mamba on the PlantDoc dataset (mean ± standard deviation).

Fold	OA (%)	Precision (%)	Recall (%)	Macro F1 (%)
Fold 1	92.53	91.72	91.48	91.60
Fold 2	92.18	91.45	91.21	91.33
Fold 3	92.76	91.94	91.68	91.81
Fold 4	92.41	91.63	91.39	91.51
Fold 5	92.19	91.46	91.15	91.30
Mean ± Std	**92.41 ± 0.24**	**91.64 ± 0.20**	**91.38 ± 0.21**	**91.51 ± 0.21**

Additionally, Bootstrapping (*n* = 1,000 resamples with replacement from the test set) yields the following 95% confidence intervals: OA = [91.87%, 93.21%], Precision = [91.02%, 92.58%], Recall = [90.78%, 92.31%], F1 = [90.91%, 92.47%]. These narrow intervals confirm that the reported performance is statistically stable and not sensitive to random train/test splits. The bold values denote the optimal performance.

### 4.6 Per-class performance analysis

To provide deeper insight into model behavior across the 27 classes, we report per-class Precision, Recall, and F1-score on the test set, as shown in **Table 6**.

**Table 6:** All disease categories performance by using the proposed approach on the PlantDoc dataset.

Class	Disease category	Train samples	Accuracy (%)
1	Apple leaf	82	93.8
2	Apple rust	78	97.6
3	Apple scrab	83	96.4
4	Bell pepper leaf	53	92.3
5	Bell pepper leaf spot	62	92.2
6	Blueberry	104	95.6
7	Cherry	47	94.7
8	Corn gray	64	85.1
9	Corn blight	179	96.8
10	Corn rust	106	93.2
11	Grape leaf	57	96.3
12	Grape rot	56	94.2
13	Peach	102	95.1
14	Potato early blight	108	90.5
15	Potato late blight	97	89.6
16	Raspberry	112	87.1
17	Soyabean	57	90.3
18	Squash powdery mildew	124	86.8
19	Strawberry	88	92.7
20	Tomato early blight	79	92.9
21	Tomato leaf	55	91.4
22	Tomato bacterial spot	101	90.8
23	Tomato late blight	101	94.4
24	Tomato mosaic	44	94.7
25	Tomato yellow	70	97.2
26	Tomato mold	85	87.3
27	Tomato Septoria	140	93.5

The per-class analysis (**Table 6**) reveals substantial performance variation across the 27 classes. Highsample categories generally achieve stronger F1-scores, whereas rare categories with limited training instances tend to underperform, corroborating the insufficient-sample-diversity error type identified in Section 4.4.

### 4.7 Computational efficiency analysis

To substantiate the claim of a lightweight design suitable for edge deployment, we benchmark all comparison models on four computational metrics: Parameters (M), GFLOPs, and Inference Time (ms/image, RTX 4090, batch=1). The results are summarized in **Table 7**.

**Table 7:** Computational efficiency comparison of the proposed improved Vision Mamba and state-of-the-art models.

Model	Params (M)	GFLOPs	Inf. Time (ms)
U-Net	31.0	8.1	9.2
MobileNetV3	27.9	0.7	3.1
DenseNet-121	28.5	7.4	8.8
EfficientNet-B4	31.2	8.2	9.5
Vision Transformer	86.4	55.4	28.3
DeiT	86.4	55.4	27.9
PVTv2	44.2	14.6	12.4
Swin Transformer	87.8	15.4	13.8
Baseline Vision Mamba	30.8	6.8	7.9
VMamba	50.3	11.2	10.6
Improved Vision Mamba (Ours)	**31.4**	**7.2**	**8.4**

The bold values denote the optimal performance.

The proposed improved Vision Mamba maintains competitive computational costs: 31.4 M parameters and 7.2 GFLOPs. This represents only a 12.3% increase in parameter count over MobileNetV3 (27.9 M) and is significantly lower than Swin Transformer (87.8 M) and ViT (86.4 M). Inference time is 8.4 ms/image, fast enough for real-time edge inference in precision agriculture scenarios. The modest overhead relative to the baseline Vision Mamba (30.8 M/6.8 GFLOPs) is attributable to the MFFM skip connections and the ACAM FFN bottleneck, both of which are designed with lightweight constraints (384 channels, reduction ratio *r* = 16). These results support the feasibility of deploying the proposed model on edge computing devices in agricultural fields.

## 5 Discussion

The experimental results on the PlantDoc dataset confirm that the proposed improved Vision Mamba model achieves superior performance in plant disease image classification compared with mainstream CNNs, transformer-based architectures, and recent SSM baselines, with an overall accuracy of 92.67% on the test set. This performance advantage stems from the rational integration of Mamba’s inherent sequence modeling capability with three customized improvement modules, which addresses the core challenges of plant disease image classification: fine-grained local lesion feature extraction, global contextual information capture, and training stability on limited data.

The proposed model advances the state of the art through three interconnected innovations that collectively address the limitations identified in existing approaches. First, the MFFM represents a departure from the single-scale feature processing of both CNNs and standard Vision Mamba by explicitly aggregating multiresolution lesion representations (texture at 28×28, shape at 14×14, and semantics at 7×7) into a unified feature map. Unlike the skip connections in DenseNet (Huang et al., 2017) that simply concatenate features, the MFFM employs resolution unification via upsampling and max-pooling with a residual addition of the 12th-layer backbone output, enabling complementary aggregation across spatial scales rather than mere feature concatenation. Second, the ACAM extends conventional channel attention mechanisms by integrating both global average pooling and global max pooling to capture both average channel statistics and local maximum responses (e.g., lesion core regions), followed by a lightweight two-layer FFN with a reduction ratio of *r* = 16 that achieves background suppression with minimal parameter overhead. This design specifically targets the complex field backgrounds in the PlantDoc dataset (soil, weeds, uneven illumination), a challenge that generic SSM backbones (Zhu et al., 2024; Liu et al., 2024) do not explicitly address. Third, the LRC introduces a lightweight 1×1 convolution in the residual path that reduces channel dimension from 768 to 384 with only 294K parameters, establishing a direct gradient transmission path that alleviates vanishing gradients in the 12-layer SSM backbone while training on the small-scale PlantDoc dataset (2,572 images). Unlike standard residual connections that use identity mapping, the LRC’s lightweight convolution provides dimension matching with controlled parameter growth, which is critical for preventing overfitting on limited training data. The synergistic combination of these three modules yields a 5.33% absolute accuracy improvement over the baseline Vision Mamba, demonstrating that architectural innovations specifically tailored to plant disease imagery can substantially enhance generic SSM backbones.

### 5.1 SSM family comparison

Under our unified PlantDoc protocol, the baseline Vision Mamba achieves 87.34% accuracy, while VMamba improves to 89.12% through its cross-scan (SS2D) mechanism that enhances 2D spatial continuity. However, VMamba still lacks task-specific modules for multi-scale lesion aggregation and background suppression. Our improved Vision Mamba closes this gap by adding the MFFM (which fuses texture, shape, and semantic features at 14×14) and the ACAM (which selectively amplifies disease-relevant channels), yielding a further 3.55% absolute gain over VMamba and 5.33% over the plain Vision Mamba. This progression demonstrates that generic SSM backbones benefit substantially from architectural modifications tailored to the target visual domain.

Unlike CNNs such as DenseNet-121 and EfficientNet-B4, which rely on fixed receptive fields and struggle to model long-range dependencies between lesion regions and plant organs, the Vision Mamba’s selective SSM enables efficient encoding of sequential spatial information, allowing the model to capture the positional and morphological correlations of discrete lesions. The MFFM further compensates for the deficiency of single-scale feature representation in the original Vision Mamba, aggregating low-level texture features and high-level semantic features to enhance the recognition of fine-grained plant disease categories with subtle morphological differences.

The ACAM in the improved model dynamically assigns weight coefficients to different feature channels, effectively suppressing redundant background information such as soil, weeds, and dense foliage in field-acquired PlantDoc samples and highlighting disease-related feature channels. This mechanism explains the model’s stronger resistance to complex background interference compared with MobileNetV3 and ViT, as reflected in the error analysis where complex background interference accounts for only 29.7% of misclassifications. The LRC module alleviates the gradient vanishing problem in the deep Vision Mamba network, ensuring stable feature learning during 100 epochs of training and maintaining the model’s generalization ability on the small-scale PlantDoc dataset (2,572 total images). In contrast, transformer-based models such as ViT and DeiT suffer from high computational complexity and insufficient inductive bias, leading to suboptimal performance on small-scale plant disease datasets.

The ablation study results demonstrate the synergistic effect of the three core improvement modules, with each module contributing to the performance enhancement of the baseline Vision Mamba and the combination of all three modules leading to a 5.33% absolute improvement in overall accuracy. The MFFM yields the most significant single-module improvement (2.15%), which verifies that multi-scale feature aggregation is a key factor in improving the accuracy of plant disease classification, as plant disease symptoms exhibit distinct morphological characteristics at different spatial scales. The ACAM and LRC module play complementary roles in feature optimization and training stability, respectively, and their combination with the MFFM further amplifies the model’s feature learning capability.

Misclassification errors in the experimental results are mainly attributed to inter-class similarity of plant diseases (58.2%), which is an inherent challenge in plant disease image classification due to the high visual similarity of lesion symptoms among different disease categories on the same or related plant species. For example, early blight of potato and tomato leaf spot share similar color and shape characteristics of lesions, making it difficult for the model to distinguish their category-specific features even with advanced feature encoding capabilities. The confusion matrix (**Figure 5**) quantifies these patterns, showing that 23% of potato early blight samples are misclassified as tomato leaf spot. The second major error type is complex background interference, which is a common problem in field-acquired plant image datasets and cannot be completely eliminated by data augmentation alone. The small proportion of errors caused by insufficient sample diversity (12.1%) indicates that the proposed model has good few-shot learning ability on the PlantDoc dataset, though the performance on rare disease categories with limited training samples still has room for improvement.

### 5.2 Computational efficiency and edge deployment

Despite the accuracy gain, the proposed model maintains competitive computational costs (31.4 M parameters, 7.2 GFLOPs), which is only 12.3% higher than MobileNetV3 and significantly lower than Swin Transformer, making it feasible for edge deployment in precision agriculture. The narrow confidence intervals from Bootstrapping ([91.87%, 93.21%] for accuracy) and the sTable 5-fold cross-validation results (92.41 ± 0.24%) further support the reliability of the model in real-world deployment scenarios where training-test splits may vary.

Compared with existing plant disease detection methods based on deep learning, the proposed improved Vision Mamba model has two main advantages for precision agriculture applications. First, the model maintains high classification accuracy while having a relatively lightweight architecture, as the improvement modules adopt lightweight design principles that avoid excessive increase in model parameters and computational complexity, making it suitable for deployment on edge computing devices in agricultural fields. Second, the model’s strong feature learning capability on the PlantDoc dataset, which covers 13 plant species and 27 classes, indicates its good generalization ability for diverse plant disease types, providing a scalable solution for large-scale plant disease monitoring in precision agriculture. However, the model’s performance is currently limited to the PlantDoc dataset, and its generalization ability on other plant disease datasets with different image acquisition conditions and plant species needs to be further verified.

## 6 Conclusion

This study proposes an improved Vision Mamba network for plant disease image classification on the PlantDoc dataset, and the systematic experimental results confirm the effectiveness and superiority of the proposed method. By integrating an MFFM, an ACAM, and an LRC module into the original Vision Mamba, the model effectively combines the efficient long-range sequence modeling capability of Mamba with fine-grained feature extraction and optimization strategies, achieving an overall accuracy of 92.67%, a macro precision of 91.83%, a macro recall of 91.56%, and a macro F1-score of 91.70% on the PlantDoc test set under a unified training protocol. These results outperform all comparison models including classical CNNs, lightweight networks, transformer-based architectures, and recent SSM baselines, demonstrating that the improved Vision Mamba is a promising approach for plant disease image classification.

The ablation study confirms the indispensable contribution of each core improvement module: the MFFM provides the largest single-module gain (2.15% accuracy improvement), the ACAM effectively suppresses complex background interference, and the LRC ensures stable training on the limited PlantDoc dataset. The synergistic combination of all three modules yields a 5.33% absolute accuracy improvement over the baseline Vision Mamba, demonstrating that their integration produces complementary benefits that exceed the sum of individual contributions. The error analysis and confusion matrix clarify the main sources of misclassification in plant disease classification, with inter-class disease similarity being the primary factor, followed by complex background interference and insufficient sample diversity. The 5-fold cross-validation and Bootstrapping analysis confirm that these findings are statistically robust and not sensitive to random data splits.

In practical applications for precision agriculture, the proposed improved Vision Mamba model provides an efficient solution for plant disease detection with its high classification accuracy, relatively lightweight architecture (31.4 M parameters, 7.2 GFLOPs), and strong statistical robustness, laying a foundation for the development of edge-deployable plant disease monitoring systems. The research results also confirm the potential of state space models represented by Mamba in the field of agricultural computer vision, breaking the limitations of traditional CNNs and transformers in modeling spatial sequence information of plant images.

For future research, three main directions are proposed based on the limitations of the current study. First, construct a larger-scale and more balanced plant disease dataset by supplementing training samples of rare disease categories and collecting images under diverse field acquisition conditions, to further improve the model’s generalization ability and reduce misclassifications caused by insufficient sample diversity. Second, design a similarity-aware feature enhancement module for inter-class similar plant disease categories, by mining category-specific discriminative features and enhancing their representation in the model, to address the primary source of misclassification errors. Third, integrate background removal and image preprocessing algorithms into the plant disease classification pipeline, to eliminate the interference of complex field backgrounds on disease feature extraction and further improve the model’s classification accuracy in real agricultural scenarios. In addition, the proposed improved Vision Mamba model will be verified on other public plant disease datasets and applied to real-time plant disease monitoring systems in agricultural fields, to further explore its practical value in precision agriculture and promote the intelligent development of plant disease detection technology.

## Data Availability

The original contributions presented in the study are included in the article/supplementary material. Further inquiries can be directed to the corresponding author.
